# Improvement of Serum Biochemical Parameters and Hematological Indices Through α-Tocopherol Administration in Dietary Oxidized Olive Oil Induced Toxicity in Rats

**DOI:** 10.3389/fnut.2018.00137

**Published:** 2019-01-10

**Authors:** Alam Zeb, Ayaz Ali Khan

**Affiliations:** Laboratory of Biochemistry, Department of Biotechnology, Faculty of Biological Sciences, University of Malakand, Lower Dir, Pakistan

**Keywords:** oxidized olive oil, α-tocopherol, TBARS, lipid peroxidation, fatty liver, rats

## Abstract

Dietary oxidized olive oil, alone or in combination with different doses of α-tocopherol, were given to Swiss albino rats for 30 days; in order to determine its role in oxidative stress and fatty liver, induced by the oxidized olive oils. Serum biochemical parameters and hematological indices of blood were analyzed. The liver was analyzed for histopathological changes, lipid peroxidation, and polar triacylglycerols composition. Results revealed that there was a significant decline in the serum total cholesterol, triglycerides, LDL, glucose and ALT; while a significant increase occurred in the serum HDL levels through the supplementation of α-tocopherol in male and female rats. Hematological parameters were almost in the normal reference range in the groups that were fed α-tocopherol, alone or in combination with oxidized oil, while being significantly altered by the oxidized olive oil. There were acute hepatitis and necrosis in the liver with no fatty changes after feeding with oxidized olive oil, along with varying doses of α-tocopherol. Higher amounts of polar compounds were present in female rats (15.2–93.1 μg/g) compared to male rats (12.2–82.3%) that correspond to the supplementation of α-tocopherol in combination with oxidized oil. Lipid oxidation in liver was minimized by tocopherol, while an increase occurred in the accumulation of oxidized lipids in the liver. These findings revealed that tocopherol is beneficial against the oxidized oil induced biochemical and hematological changes and lipid peroxidation but causes fatty accumulation in the liver. Therefore, the role of tocopherol in patients with fatty liver disease may be considered, as tocopherol may increase the chance of survival.

## Introduction

Thermally oxidized lipids are considered one of the crucial components in modern-day food preparation. Oils and fats, when thermally oxidized, produce primary oxidized triacylglycerols (1), which are usually absorbed by the food materials and enter the human body. Oxidized lipids from fried diet thus become part of the chylomicron in the blood (2). From the chylomicron, these oxidized lipids may reach several parts of the body including the liver. In the liver, the non-alcoholic fatty liver is one of the major conditions contributed by oxidized lipids, even when the olive oil is used for frying (3, 4). Hepatitis and chronic adenoma have been caused by oxidized lipids. It also enhanced the expression of vascular endothelial growth factor in human colorectal tumor cells, thereby promoting cancer (5). In rats, thermally oxidized lipids have been found to significantly accelerate hepato-carcinoma (6). A significant increase in serum cholesterol, low-density lipoproteins (LDL) and triglycerides (TG) have been observed in experimental animals, which was caused by thermally oxidized fats and was a major contributor of atherosclerosis (7). The human atherosclerotic plaque has been found to contain both oxidized lipids and relatively large amounts of α-tocopherol and ascorbic acid (8). Several dietary supplements have been found to reduce the negative effects caused by oxidized lipids, such as tomato (9), Seabuckthorn seed powder (10), seed oil (11), and amino acids (glycine & glutamic acid) (12).

Alpha-tocopherol is a natural antioxidant present in the cells of plants and animals. When rats were supplemented with diets containing α-tocopherol, it was found to maintain the cellular redox status, especially, by preserving the activities of important enzymes; such as superoxide dismutase, catalase, glutathione peroxidase, glutathione reductase, as well as, glutathione and vitamin E levels. The levels of peroxides, hydroxyl radical and hydrogen peroxides of lipids significantly decreased (13). In combination with catechin or quercetin, α-tocopherol plays a synergistic antioxidant role in the liposome oxidation (14). The dietary supplementation of α-tocopherol helps to stabilize the membrane-bound lipids in the muscles and tissues of broiler chickens and pigs (15). However, when thermally oxidized sunflower oil was fed to the chicks, it reduced the α-tocopherol status and increased the susceptibility of the tissues to the lipid peroxidation (16). Similarly, Sheehy et al. (17) showed that diets containing heated vegetable oils had a significantly reduced serum α-tocopherol status in chicks. It also produced changes in the fatty acid composition of their muscle lipids. These authors showed that oxidized lipids were responsible for increased susceptibility of lipid peroxidation. There is, however, a lack of information about the effects of α-tocopherol on the fatty liver produced by oxidized lipids. This study aims to reveal the interactions of α-tocopherol during fatty liver induced by oxidized lipids in rats, for the first time.

## Materials and Methods

### Materials

Fresh olive oil was purchased from the local market in Chakdara, Lower Dir, Khyber Pakhtunkhwa, Pakistan. The olive oil was selected based on its high contents of oleic acid (4) and high thermal stability. Serum biochemical test kits were brought from Merck (Merck, Germany) and Human (Human Diagnostics, Germany). Methanol, α-tocopherol, isopropanol was obtained from Sigma-Aldrich (Germany). All other chemicals and reagents were of analytical grade.

### Thermal Treatment of Oil

Olive oil was heated for 5 h at 100°C on a hot plate, and soon after, was stored in the refrigerator at −20°C to avoid any further alteration till its use. The oleic acid composition of the control and oxidized olive oils were 77.9 and 81.4 mg/100 g, respectively. The characteristics of normal and oxidized olive oil have been reported in our recent work (4).

### Animals Feeding

A total of 48 Swiss albino male and female rats (10–11 weeks old, average weight 200 g) were selected after rearing in a bio-park of the University of Malakand. The experiments were approved by the ethical committee, the graduate studies committee, and the advanced studies and research board of the University of Malakand for proper care and experimentation. The composition of the basal casein diet given to rats consisted of casein (22%), sucrose (69.5%), corn oil (4.5%) and a salt mixture IV (4.0%). Rats were divided into 16 groups; 8 males and 8 female groups. Each group consisted of three animals. Two groups (male & female) were fed a normal diet without any treatment. The dosing scheme is given as under with F, M, T, and O representing female, male, tocopherol, and oxidized oil, respectively.

FC group was fed a normal diet; FO group was fed oxidized oil (2 g/kg body weight); FT1 group was fed tocopherol (100 mg/kg body weight); FT2 group was fed tocopherol (200 mg/kg body weight); FT3 group was fed tocopherol (300 mg/kg body weight); FTO1 group was fed oxidized oil (2 g/kg body weight) and tocopherol (100 mg/kg body weight); FTO2 group was fed oxidized oil (2 g/kg body weight) and tocopherol (200 mg/kg body weight) and FTO3 group was fed oxidized oil (2 g/kg body weight) and tocopherol (300 mg/kg body weight). The male rats were fed a similar dosing scheme. Feeding was carried out regularly for 1 month and at the end of the period, the rats were humanely killed, and blood samples collected.

### Biochemical Parameters

The serum was used for the analyses of biochemical parameters, such as total cholesterol, total triglycerides assay kits (Merck, Germany), while HDL-c, LDL-c, ALT, and glucose assay kits were of Human (HUMAN Diagnostics, Germany).

### Hematological Parameters

Three milliliters of whole blood samples were transferred to heparinized tubes. Whole blood was used for the analyses of total red blood cells, hemoglobin concentration, hematocrit value, mean corpuscular volume, mean corpuscular hemoglobin, mean corpuscular hemoglobin concentration, total leukocyte count, neutrophils, eosinophil, lymphocytes, and platelets count by employing a fully automated blood hematology analyzer (SYS MIX, Japan).

### Histopathological Examination of Liver

After killing the rats, their liver was isolated and preserved in a formalin solution. Tissues sectioning were made, stained and histopathological examination was done as described earlier (11). Microscopic slides were observed under a light microscope, model No. M 7000 D (SWIFT, Japan) and the pictures were taken with a digital camera mounted on a microscope with the resolution of 1.3 MP.

### Extraction of Liver Lipids

Lipids were extracted using the method of Folch et al. (18). Briefly, 1 g of each homogenized liver sample was put into triplicates of 250 mL volumetric flasks and extracted with chloroform-methanol mixture (2:1). The extracted lipids were analyzed for lipid peroxidation and polar triacylglycerols.

### HPLC Analyses of Polar Compounds

Polar or oxidized compounds in the liver lipids were determined using the HPLC-DAD method reported previously (19). Twenty microliters of the extracted lipids sample were dissolved in acetone-isopropanol and filtered with Agilent 0.45-micron PTFE filter (Agilent Technologies, Germany) into 2 mL HPLC vial. The separation was carried out using Agilent Zorbax Rapid Resolution C-18 (100 × 4.6 mm, 3.5 μm) column at 25°C using isopropanol: methanol (18:82, v/v) at a flow rate of 1 mL/min. The chromatogram was resolved at 210 nm using Agilent Chemstation.

### Lipid Peroxidation in Liver

Lipid peroxidation was studied in the extracted liver lipids using a sensitive spectrophotometric method, as thiobarbituric acid reactive substances (TBARS). The method uses the reaction of malondialdehyde (MDA) and thiobarbituric acid (TBA) in the glacial acetic acid. The extracted lipids (200 mg) were mixed with 5 mL of glacial acetic acid-water (1:1, v/v) and shaken for 1 h. The samples were then centrifuged at 4,000 rpm for 10 min and absorbance was measured at 532 nm (20).

### Data Analyses

The data were expressed as mean with standard deviation (SD) of replicate measurements using GraphPad Prism version 7.01 (GraphPad Software, Inc, 2016). The analysis of variance (ANOVA) was performed for significant differences between mean values vs. control at different *p* values and significance was represented by different letters where appropriate.

## Results

### Serum Biochemistry

**Figure 1:**
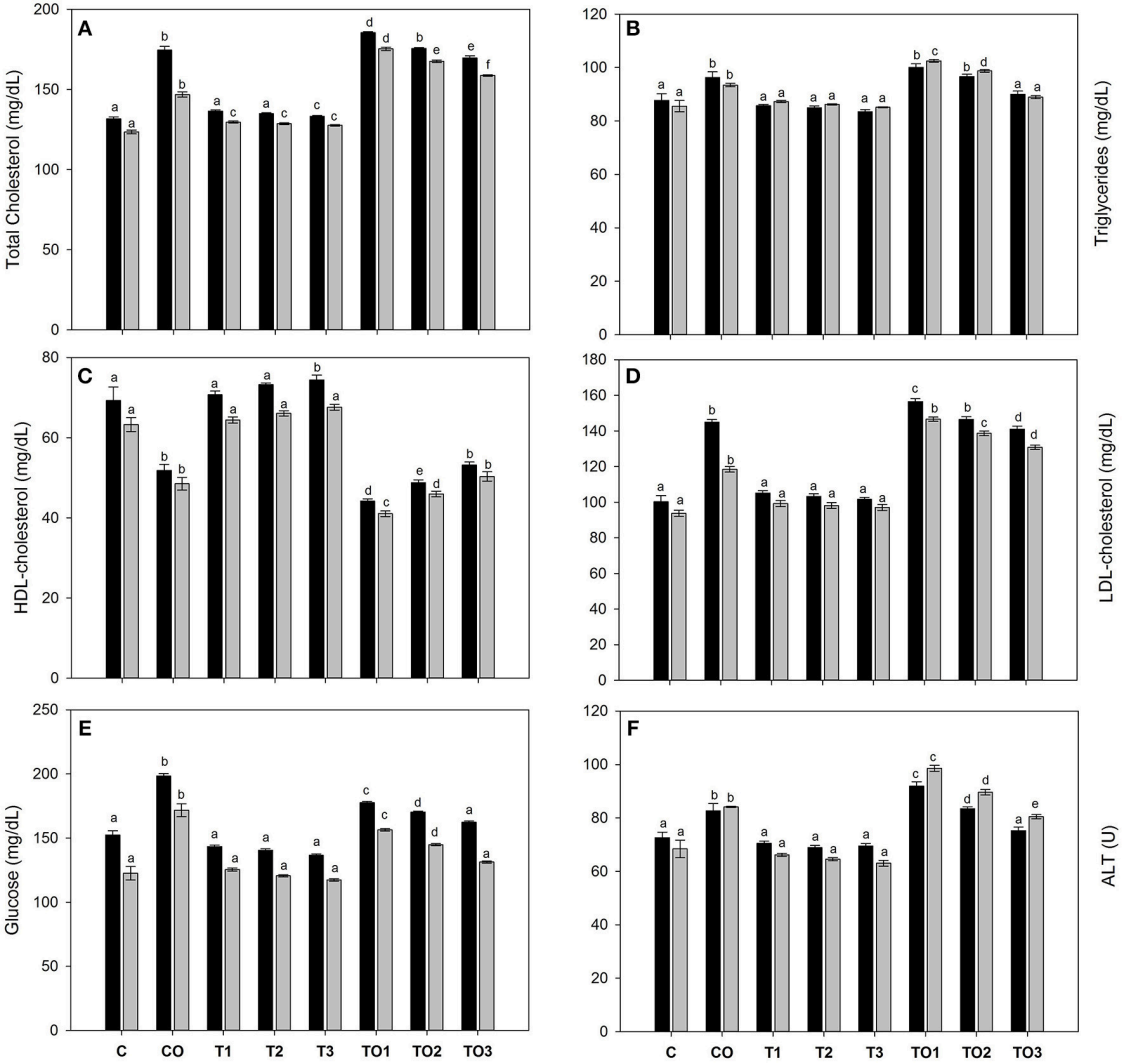
Effects of α-tocopherol alone and in combination with thermally oxidized olive oils on the serum biochemical parameters of rats, **(A)** total cholesterol, **(B)** triacylglycerols, **(C)** HDL-c, **(D)** LDL-c, glucose **(E)**, and **(F)** ALT. Data are means ± SD of *n* = 3 per group. Different letters (a–f) on each male and female group represent significant at *p* < 0.001. Black bars are male groups, while gray bars are female groups.

The supplementation of α-tocopherol alone had a positive effect on the serum biochemical parameters as shown in Figure 1. However, the total cholesterol amounts increased with supplementation of oxidized olive oil. The co-administration of α-tocopherol and oxidized oil significantly (*P* < 0.001) reduced the total cholesterol amounts in both male and female rats (Figure 1A). Similarly, there was no significant change in the serum triglycerides with α-tocopherol alone, but it increased in the CO group. The increase in triglycerides was recovered by the co-administration of α-tocopherol and oxidized oils (Figure 1B). The administration of α-tocopherol alone significantly increased the serum HDL-cholesterol levels, while it significantly declines by oxidized oil. The co-administration of α-tocopherol improved the levels of HDL-c (Figure 1C). The LDL-c was not affected by α-tocopherol alone but was significantly increased by oxidized oils. The α-tocopherol was beneficial in improving the serum LDL-c levels (Figure 1D). Similar observations were also reported in the case of serum ALT levels. The serum glucose was also normalized by the supplementation of α-tocopherol in combination with oxidized olive oil. These results suggest that α-tocopherol was beneficial for improving serum biochemical parameters. With exception of serum triglycerides, all biochemical parameters were lower in female rats as compared to male. The effects of tocopherol supplementation revealed significant effects in female rats.

### Hematological Indices

Oxidized oil was given to male rats at a dose rate of 2 g/kg along with α-tocopherol at the dose rate of 100, 200, and 300 mg. The amounts of RBC were significantly decreased upon supplementation of oxidized oil and α-tocopherol (Table 1). The WBC and lymphocytes increased significantly when oxidized oil and α-tocopherol were supplemented. However, with an increase of α-tocopherol, normalization of these hematological values were recorded. The amount of Hb, HCT, and PLT were significantly decreased when oxidized oil was used and was normalized by the increase of α-tocopherol. There were no significant effects on the MCV and MCH values. These results concluded that supplementation of α-tocopherol has beneficial effects on hematological parameters in rats.

**Table 1:** Effects of α-tocopherol alone or in combination with thermally oxidized olive oils on the hematological parameters of rats.

**Parameters (Unit)**	**Male rats**								**Female rats**							
	**C**	**CO**	**T1**	**T2**	**T3**	**TO1**	**TO2**	**TO3**	**C**	**CO**	**T1**	**T2**	**T3**	**TO1**	**TO2**	**TO3**
RBC (×10^6^/μL)	7.7 ± 0.6	5.8 ± 0.1	7.83 ± 0.3	8.26 ± 0.3	8.56 ± 0.1	6.2 ± 0.1	7.1 ± 0.2	7.83 ± 0.2	7.13 ± 0.3	5.9 ± 0.05	6.93 ± 0.2	7.66 ± 0.2	7.86 ± 0.1	5.93 ± 0.2	6.7 ± 0.3	7.1 ± 0.3
WBC (×10^3^/μL)	9.13 ± 0.4	10.1 ± 0.6	8.93 ± 0.2	8.26 ± 0.2	8.0 ± 0.1	11.0 ± 0.3	10.1 ± 0.2	8.9 ± 0.2	8.4 ± 0.3	10.0 ± 0.3	8.9 ± 0.2	8.3 ± 0.1	8.6 ± 0.1	11.1 ± 0.4	10.4 ± 0.3	9.0 ± 0.2
Hb (%)	14.6 ± 0.7	12.1 ± 0.3	16.13 ± 0.2	16.97 ± 0.3	17.7 ± 0.2	13.32 ± 0.1	14.63 ± 0.4	16.4 ± 0.3	13.4 ± 0.5	12.0 ± 0.2	15.0 ± 0.2	16.2 ± 0.3	17.1 ± 0.2	13.2 ± 0.2	14.3 ± 0.3	15.7 ± 0.3
HCT (%)	45.5 ± 0.4	39.7 ± 0.6	47.3 ± 0.3	48.2 ± 0.3	49.7 ± 0.2	41.9 ± 0.4	43.6 ± 0.4	45.4 ± 0.3	43.5 ± 0.6	37.0 ± 0.2	46.6 ± 0.4	47.9 ± 0.3	48.8 ± 0.2	39.8 ± 0.4	42.5 ± 0.4	44.5 ± 0.1
MCV (fL)	56.1 ± 3.3	50.9 ± 0.7	59.2 ± 0.3	60.3 ± 0.3	61.7 ± 0.3	53.9 ± 0.7	55.8 ± 0.4	58.8 ± 0.3	51.9 ± 2.2	48.0 ± 0.9	58.0 ± 0.3	59.4 ± 0.3	60.0 ± 0.2	51.9 ± 0.4	54.0 ± 0.4	57.0 ± 0.3
MCH (pg)	18.0 ± 1.0	15.0 ± 1.0	19.8 ± 0.5	21.5 ± 0.5	23.3 ± 0.4	18.9 ± 0.4	20.1 ± 0.4	21.9 ± 0.3	16.0 ± 1.0	15.0 ± 1.0	18.8 ± 0.5	20.4 ± 0.4	21.8 ± 0.6	16.4 ± 0.4	17.8 ± 0.5	19.2 ± 0.3
MCHC (g/dL)	39.0 ± 1.0	35.5 ± 0.3	31.1 ± 0.4	32.7 ± 0.6	34.0 ± 0.3	30.1 ± 0.4	31.8 ± 0.7	33.3 ± 0.5	37.3 ± 1.2	34.0 ± 0.5	30.1 ± 0.4	31.1 ± 0.4	32.2 ± 0.3	28.9 ± 0.5	30.9 ± 0.7	32.4 ± 0.3
Lymphocytes (%)	71.3 ± 4.5	84.0 ± 1.7	71.3 ± 0.3	70.1 ± 0.9	68.5 ± 0.4	74.2 ± 0.3	73.1 ± 0.2	71.2 ± 0.2	75.3 ± 2.0	83.0 ± 3.2	72.0 ± 0.3	71.2 ± 0.1	70.3 ± 0.2	75.5 ± 0.3	74.3 ± 0.3	71.6 ± 0.2
PLT (×10^3^/μL)	304.0 ± 11	402.0 ± 9.0	455.3 ± 10.6	509.0 ± 16.5	600.0 ± 11.0	342.0 ± 12.7	417.0 ± 12.0	506.0 ± 0.3	290.0 ± 7.5	392.0 ± 5.5	447.7 ± 8.5	512.3 ± 11.0	624.0 ± 10.1	311.7 ± 10.5	426.0 ± 14.1	527.0 ± 11.1

*Values are expressed as Mean ± SD of n = 3. C, is control; CO, is control oxidized; M, male; F, female; T, tocopherol; O, oxidized olive oil, and 1, 2 and 3 are the grams of the olive oil fed to rats. RBC, red blood cells; Hb, hemoglobin; HCT, hematocrit; MCV, mean corpuscular volume; MCH, mean corpuscular hemoglobin; MCHC, mean corpuscular hemoglobin concentration; WBC, white blood cells; PLT, platelets*.

### Histopathological Examination

**Figure 2:**
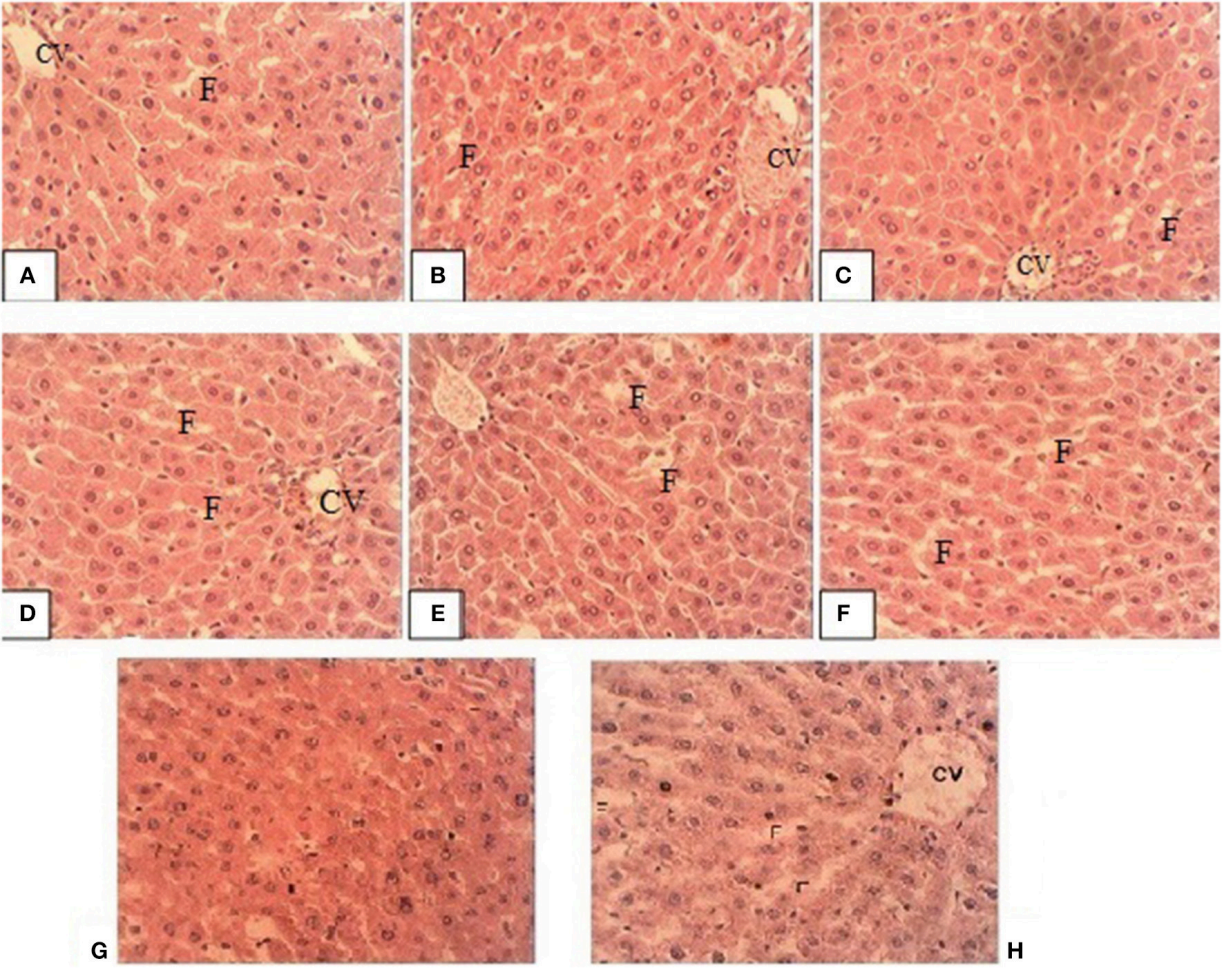
Representative liver sections of male rats **(A)** TO1, **(B)** TO2, **(C)** TO3, **(D)** T1, **(E)** T2, **(F)** T3, **(G)** control and **(H)** oxidized olive oil group. CV is central vein and F is fats accumulated.

Figure 2 shows the liver sections of the male rats fed both oxidized olive oil, alone or in combination with different doses of tocopherol. In both the male and female rats, in the liver, there was normal lobular architecture with no inflammation. Few inflammatory areas can be seen in centrilobular regions, which was a sign of toxicity of tocopherol. These changes were slightly increased from lower to higher doses, i.e., 100–300 mg/kg. In both male and female rats, liver showed piecemeal necrosis with acute hepatitis. The overall architecture was preserved with no fatty changes. Fatty infiltration, marked as F, was obvious in rats fed oxidized oil and tocopherol in combination. These results showed that increased tocopherol supplementation produced negative effects on the liver by affecting its structure and function.

### Polar Compounds in Liver

**Figure 3:**
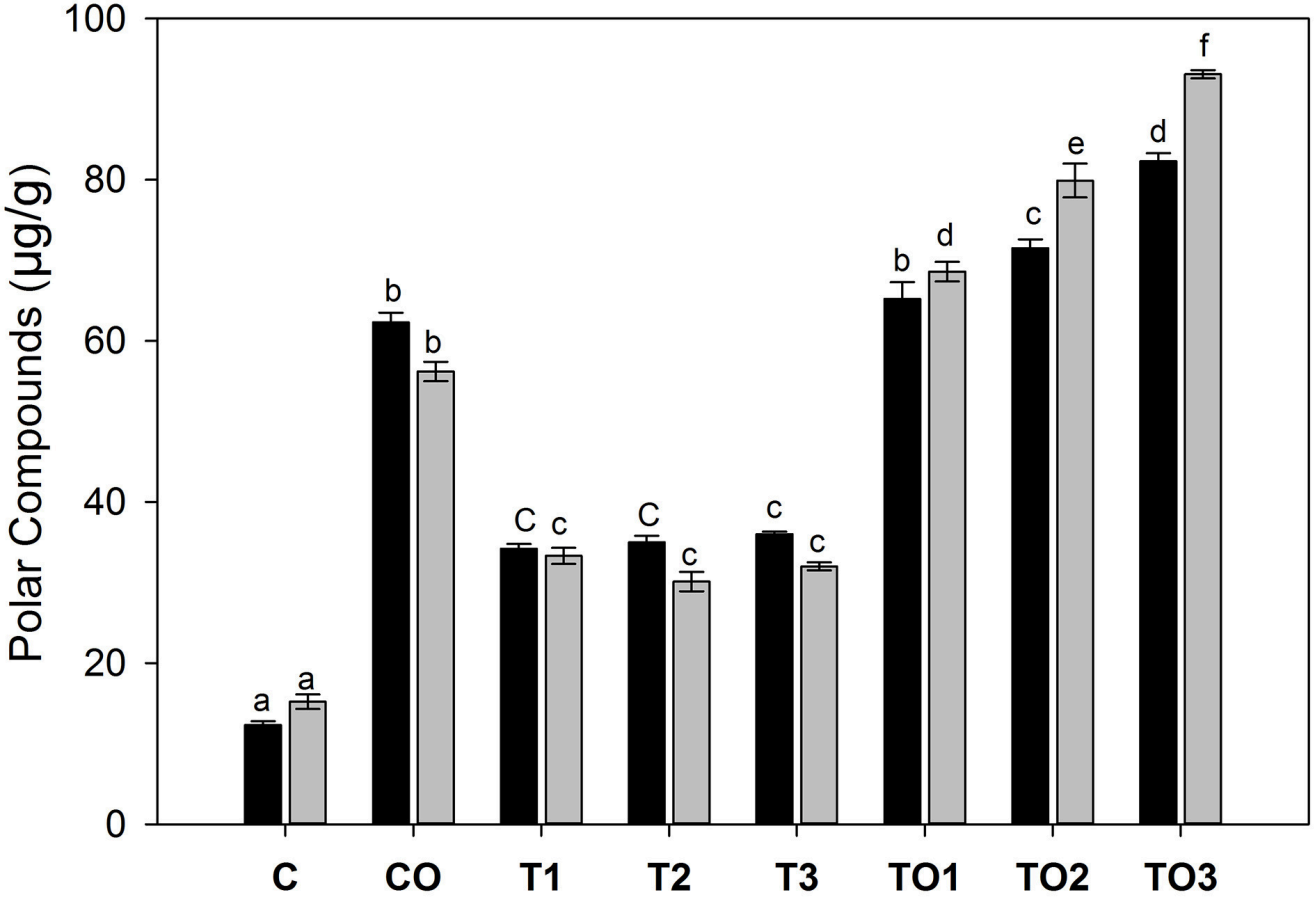
Effects of supplementation of α-tocopherol alone or in combination with oxidized olive oil on the amount of polar compounds. Different letter (a–f) in the male or female rats represents significance at *p* < 0.05 (*n* = 3). M, F, T, C, and O are the abbreviations for male, female, tocopherol, control and oxidized lipids. Statistical comparison was carried out within the same male and female group independently. Black bars are male groups, while gray bars are female groups.

The extracted lipids were analyzed by HPLC-DAD for polar compounds, especially, polar triacylglycerols. It was found that because of the polar nature, all compounds were eluted at the first 3 min of elution. The amounts of normal triacylglycerols were very low as compared to the polar compounds, thus, a relatively large amount of polar lipids was obtained. As shown in Figure 3, female rats were affected more by tocopherol as compared to its corresponding doses of male rats. In male rats, the amount of polar triacylglycerols were 34.2, 35.0, and 36.1 μg/g that corresponds to 100, 200, 300 mg doses of tocopherol, respectively. At the same dose rates, the values of polar compounds were 33.3, 30.1, and 32.0 μg/g in female rats. A significantly higher number of polar compounds were found in female rats (15.2–93.1 μg/g) compared to male rats (12.2–82.3%) that correspond to the 100–300 mg of α-tocopherol in combination with oxidized oil supplementation. These results showed that supplementation of large amounts of α-tocopherol (100–300 mg) in combination with oxidized lipids increased the number of polar compounds in the liver.

### Lipid Poxidation in the Liver

**Figure 4:**
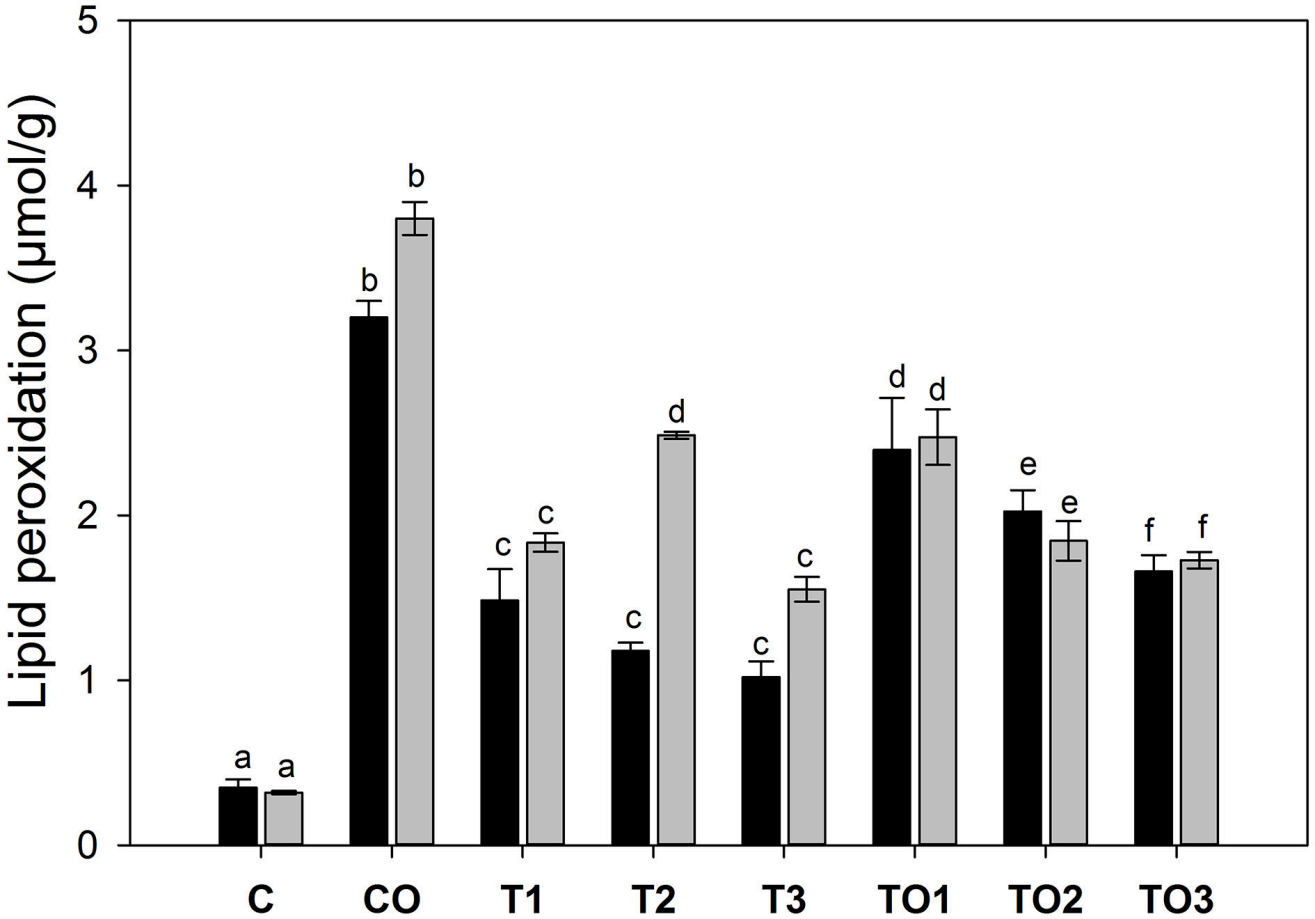
Effects of supplementation of α-tocopherol alone or in combination with oxidized olive oil on the lipid peroxidation in the liver. Different letter (a–f) in the male or female rats represents significance at *p* < 0.05 (*n* = 3). M, F, T, C, and O are abbreviations for male, female, tocopherol, control and oxidized lipids. Statistical comparison was carried out within the same and female group independently. Black bars are male groups, while gray bars are female groups.

The TBARS value is one of the key parameters for the determination of total lipid peroxidation in the tissue and is different than the polar triacylglycerol (TAGs) analyzed by HPLC. Figure 4 shows the effects of supplementation of tocopherol alone or in combination with oxidized olive oil on the lipid peroxidation in the liver. It is clear that in control male rats, the lipid peroxidation decreased significantly (*p* < 0.05) with the increasing dose amount. There was the relatively double the amount of lipid peroxidation as TBARS of 2.48 μmol/g of the tissue compared to its corresponding control male rats (1.48 μmol/g). The increase of tocopherol at 300 mg significantly decreased the amount of TBARS level equivalent to the amount of control samples. In female rats, the amount of TBARS was higher in control than its corresponding doses in male rats. There was a significant increase in the amount at the dose of 200 mg in control female rats, which was found to decline by increasing further tocopherol to 300 mg. The supplementation of thermally oxidized olive oil and tocopherol increased the levels of TBARS to 2.47 μmol/g at the dose rate of 100 mg. A further increase in the tocopherol showed a decrease of TBARS values.

## Discussion

Thermal oxidation of olive oils produces significant amounts of polar TAGs (21). The heating of olive oil at 100°C oxidized hydroxytyrosol derivatives and α-tocopherol and thus became prone to rancidity (22). The polar compounds, when consumed, produce significant negative effects on serum biochemistry in the experimental animals. In the present work, we have observed that tocopherol, when supplemented alone or in combination with oxidized olive oils, had a beneficial effect by normalizing the values of serum cholesterol, TG, HDL-c, glucose and ALT; and decreased the amount of LDL-c. The effects tocopherol supplementation on biochemical parameters were more pronounced in female rats as compared to male rats. Previous studies showed that oxidized fats are present in foods. After absorption, it increases serum cholesterol level and may lead to atherosclerosis (23). Vitamin E administration in small dose decreases the atherogenesis and also decreases atherogenic indices (24). Vitamin E reduces cholesterol level in rabbits (25, 26) and mice (27). In atherosclerosis, there was a high blood triglyceride level, and oxidized dietary fats play a role in the progression of atherosclerosis (28), which confirm our findings. Similarly, vitamin E was checked for its ability to decrease blood glucose and was found that it can significantly decrease blood glucose level (29) by improving insulin resistance. It had been observed that oxidative stress induced by rancid oils leads to liver injury, which caused increased in the ALT level (30) and could be minimized by tocopherol (31). Thus, tocopherol was beneficial for serum biochemistry.

Oxidized oil was given to male rats at a dose rate of 2 g/kg along with α-tocopherol at the dose rate of 100–300 mg; RBC, Hb level, HCT value, MCV, MCH, MCHC, granulocytes, and platelets count were low, when oxidized olive oil was given along with α-tocopherol in small dose. As the dose of α-tocopherol was raised, these values were normalized. Hemoglobin level, RBC, and MCV were decreased after feeding rats with different fresh and fried oils (32). After administration of vitamin E at different dosage levels, an increase was found in RBC, Hb level, MCV and granulocytes, and MCH were decreased (33). These results indicate that supplementation of tocopherol alone, and in combination with oxidized olive oil, were beneficial for hematological parameters.

The histological studies revealed that thermally oxidized olive oils caused significant negative changes in the structure and functions of the liver, which was enhanced by supplementation of tocopherol. Tocopherol alone has no significant effects on the liver, with the exception of causing inflammation. The literature showed that thermally oxidized edible oils lead to the histological alteration in liver (12, 34). Our results are in agreement with the previous findings that vitamin E supplementation to rats after intoxication with sodium fluoride, did not overcome the toxicity in liver tissues completely (35). This means that oxidative stress produced by thermally-oxidized lipids and other toxicants are not completely ameliorated by tocopherol but were effective enough against the full toxic spectrum. A previous study (36) showed that consumption of olive oil significantly decreases triglycerides contents in the rat liver, but does not provide sufficient antioxidant activity. In the present study, oxidized oils with fewer amounts of unsaturated fatty acids were able to accumulate oxidized lipids in the liver.

It has been found that supplementation of tocopherol and oxidized lipids, in combination or alone, increased the polar compounds in the liver in all treated rats. In the earlier experiments (4), it was observed that thermally-oxidized lipids cause fatty infiltration in the liver of the selected animals. The increase in the amount of polar lipids may be responsible for fatty liver and thus increased the dose of tocopherol may be one responsible factor. The primary oxidation of lipids may result in the formation of secondary oxidation products, which is responsible for several detrimental effects. The TBARS is thus the measure of secondary lipid peroxidation. Lipid peroxidation is one of the important parameters for the determination of oxidative stress in selected experimental subjects (37). The level of lipid oxidation in a meal or supplement significantly influences the markers of oxidation and inflammation in the liver (38). Together with TBARS of the liver and oxidative stress markers of serum, can be a marker for liver steatosis (39). In the present study, it was observed that the supplementation of tocopherol has been found to decrease the level of TBARS. There was a strong negative correlations coefficient of *R*^2^ = 0.8691 & *R*^2^ = 0.9999 between the tocopherol amount and the lipid peroxidation in female and male rats, respectively. This shows that tocopherol may hinder the production of secondary lipid peroxidation in the liver but cause the fatty accumulation in the liver. Previous studies showed that vitamin E reduces oxidative stress and improve insulin action (40). The tocopherol supplementation produces significant effects in the lipid peroxidation and polar compounds in female rats as compared to male rats. Recent studies also showed that vitamin E supplementation decreased oxidative stress and reduced collagen deposition in the visceral adipose tissues of mice, allowed the expansion of the adipocytes and increased the storage capability of tissues (41). Thus, it is important to consider, the role of tocopherol in a patient with fatty liver, as tocopherol may increase its chances.

## Conclusions

From these results, it has been concluded that oxidized olive oil leads to liver toxicity and also causes an alteration in biochemical parameters and hematological indices. Alpha-tocopherol, when co-administered alone or in combination with oxidized olive oil, lead to a decrease in toxic effects. However, tocopherol has been found to increase polar compounds in the liver, while hindering the formation of secondary lipid peroxidation. Thus, the use of tocopherol may be thoroughly monitored in patients with non-alcoholic fatty liver diseases.

## Author Contributions

AZ designed the experiments, analyzed the characteristics, performed the biochemical analysis, and wrote the manuscript. AK collected the samples and animals, performed the feeding of animals, and analyzed hematology and histology.

### Conflict of Interest Statement

The authors declare that the research was conducted in the absence of any commercial or financial relationships that could be construed as a potential conflict of interest.
